# Dr. Ramsbotham on Puerperal Fever

**Published:** 1811-10

**Authors:** John Ramsbotham

**Affiliations:** Old Jewry


### Medical and Physical Journal.

VOL. XXVI.] OCTOBER, 1811. [NO. 152.

Printed for R. PHILLIPS, by E. Hemsted, Great New Street, Fetter Lane, London.

*To the Editors of the Medical and Physical Journal.*

GENTLEMEN,

I AM truly sorry to find that the Puerperal Fever has shewn itself in the neighbourhood of London, and from the proximity of it's approaches there is reason to apprehend, that it may be imported into the metropolis, and rage epidemically, as it did about twenty years ago, when its fatality must be in the recollection of many of your readers. The danger and occasional insidiousness of this disease demand our closest attention, either to avert its effects, or even, in some cases, to ascertain its presence. The treatment, which has been of late the most successfully adopted in opposing its ravages, is so different from that of some years past, so apparently consonant to the nature of the disease shewn by dissections, and likewise so well established (at least in certain distant parts of the kingdom) by the practice of a most eminent Surgeon, Mr. Hey of Leeds, and by that of Dr. Gordon of Aberdeen, that I trust I shall be excused taking up a few pages of your widely circulated Journal on a subject interesting to every medical man.

I am, Gentlemen,

Your most obedient servant,

JOHN RAMSBOTHAM.

*Old Jewry, August 26th,* 1811.

On Wednesday, August 21st, about noon, I was requested to see, as soon as possible, a young woman dangerously ill, who had lain in a week, in a village a few miles from town. Upon my arrival, I found my patient in the last stage of Puerperal Fever, in a state indeed affording not the least hope from medical assistance; with the Hippocratic countenance, laborious respiration, low delirium, a rapid tremulous pulse, and a swelled belly. I was informed that she had been put to bed of her first child the Wednesday preceding, after a lingering hard labor, that she seemed to go on well until the Sunday following, about the middle of which day she had been seized with a considerable rigor, succeeded by heat upon the skin, quick pulse, and pain and tension of the belly. On the Sunday morning previous to the rigor, her medical attendant had very properly given her a common opening medicine, which had operated smartly, and to its effects were improperly attributed by the people about her the subsequent symptoms. The milk had not been properly secreted in the breasts, and though the child had been applied to them, the natural desire to suckle seemed inconsiderable: the abovementioned symptoms, *viz.* the quick pulse, heat upon the skin, pain and tension of the belly gradually increased in spite of blisters, salines, and opiates; the lochial discharge disappeared, a low muttering delirium came on, the urine was secreted in small quantity, and with difficulty evacuated, and I found her three days after the first attack, as above described. In the course of the following night she expired.

Leave was obtained to inspect the body on the following day, and those appearances which have been so frequently described by various authors, presented themselves to our notice. Upon making an incision into the abdominal cavity, a considerable quantity of a serous fluid, having white flakes of coagulated lymph, or a puriform substance swimming in it, escaped; some of it also remained in the pelvis. There was a vivid blush of inflammatory action upon the whole surface of the peritonæal covering of the intestinal canal, as well as upon that lining the abdominal cavity. The stomach and intestinal canal were fully inflated with an offensive gas which escaped on puncture; to this general inflation seemed owing in a great measure the size of the abdominal swelling. The omentum was shrivelled up or wasted, and in several parts of its substance a larger quantity of the above puriform matter was deposited in a more solid state. The uterus was soft and flaccid in its structure, and by no means well contracted for the time which had elapsed since delivery. The right ovarium was much enlarged in size, and was found in a state of suppuration: the left ovarium was also considerably enlarged. Both the Fallopian tubes were of a dark red, almost amounting to black. The peritonæal surface of the uterus had here and there patches of inflammatory action; its inner coat was quite black. The liver was apparently healthy, with its gall-bladder tense with bile. The chest and head were not examined.

My medical friend, who had attended this patient, informed formed me, that another woman lay dead in the village within a very short distance of the same disease, whose body he had also permission to inspect, and requested my presence. This woman was thirty-four years of age; she had been safely (apparently) put to bed on the Tuesday morning about four o'clock of her fifth child, after a remarkably easy labor; she was attacked with shivering succeeded by the usual symptoms on the following morning, (Wednesday) about two o'clock, and died on Thursday morning about five. The symptoms were violent and rapid, so that she was a corpse within forty-nine hours after delivery. The body was inspected on the Thursday evening.

Nearly similar appearances presented themselves as in the former case: There was not the quantity of serous fluid, nor of the puriform matter observed in the former case. The stomach and intestinal canal appeared equally inflated, and the redness upon the peritonæal coat was thought somewhat more vivid; our light was, however, but indifferent. The uterus was more firm in its structure, and more contracted; the ovaria were not observed to be enlarged. The liver was also healthy, and the gall-bladder turgid with bile.

Various have been the opinions of most respectable practitioners respecting the nature of that disease which bears the name (though, perhaps, by no means explicit or correct) of Puerperal Fever, and as various has been the treatment recommended and pursued; but surely, in so dangerous, and too frequently, so fatal a disease, it behoves the profession to attain some degree of certainty in their practice, if certainty be at all attainable. The difference of sentiment, which has hitherto prevailed on this subject, is, I apprehend, partly to be attributed to the natural difference of constitution and habits of life of those women whom this disease assails, and partly, to the sudden transition, from symptoms of high action and excitement, to those of irritation and debility in its progress. Hence we have two distinct sets of symptoms, those of increased action and those of irritation, running into one another, mixing with each other under a variety of forms, or rapidly following each other, thus producing those complicated and varied appearances which are described by numerous authors as different diseases, and in actual practice confounding the judgment of the most sagacious practitioner.

The most simple, and at the same time, in my opinion, the most correct mode of viewing this dangerous disease, is to consider it, at its first onset at least, as one of an active inflammatory description; in short, as nothing more or less than peritonæal inflammation variously modified, and attacking a puerperal woman under different existing circum-

 stances; stances; but afterwards, when this inflammatory action has existed in a part so delicate in structure till the system is somewhat exhausted, or till effusion into the abdominal cavity has taken place as its necessary consequence, the disease can be no longer considered in the same light; the appearances will have changed, and the symptoms present will be those of irritation, languor, and debility. If this view of the disease be at all correct, in which I think I am borne out by the first and subsequent symptoms, by the appearances on dissection, and by that treatment which has of late been successfully pursued at Leeds by the most eminent of the faculty there, and first pointed out by Dr. Gordon of Aberdeen, in his excellent Treatise on the subject; it will immediately determine our practice, and shew the necessity of watchfully looking for the attack and first onset of this dangerous disease, in times of its epidemical ravages, that we may embrace the only opportunity we possess of early checking its malignant effects.

The period after delivery at which the Puerperal Fever makes its attack, is universally acknowledged to be very uncertain; occasionally it will shew itself within the first twenty-four hours; most commonly about the third or fourth day; most frequently within the first week, and now and then after that period: it is, however, a general observation, that the more early after delivery it assails the patient, the more rapid is its progress, and the more dangerous its effects. Its commencement is usually announced by more or less of rigor; sometimes this symptom is scarcely observable, the disease making an insidious progress; at others it is violent. Both before and after the rigor the pulse is unusually quick, and afterwards acquires a degree of velocity peculiar to this disease. Indeed in those cases in which there has been no marked rigor, the pulse is preternaturally quick, and soon becomes rapid. In some instances, the patient, when apparently going on well, is suddenly attacked with acute permanent pain in the lower part of the abdomen, which presently becomes tender to the touch, or painful upon motion, accompanied with frequent respiration, quick pulse, and shortly after by tension of the belly with pain upon pressure. The pain at first is described as confined to a particular part, but it soon becomes general over the whole belly: if relief be not speedily obtained by the means of art, many of the above symptoms regularly increase in violence; the pulse becomes more hurried, and is diminished in strength in proportion to its rapidity; the abdomen grows more tender and tense; respiration is performed frequently and laboriously; the tongue becomes furred or glossy; the urinary secretion is diminished in quantity, and is evacuated with difficulty; the countenance countenance has a strongly marked dejectedness and anxiety; a low delirium is observed at intervals, and death soon closes the scene. In acute cases the disease will prove fatal within twenty-four hours from its first attack; in common ones in four or five days, though some are protracted beyond that period. In a very few instances suppuration has been known to have been effected through the abdominal parieties, after which recovery has ensued. If the disease occurs after the secretion of milk has been established, that nutritive fluid is quickly absorbed from the breasts, and the secretion afterwards becomes suppressed. The common lochial discharges also disappear after the disease has commenced its career.

The symptoms preceding recovery are, a diminution in the velocity of the pulse, of the pain and tension of the belly, refreshing sleep, and change of posture.

The diagnostic symptoms are, more or less of rigor in a puerperal woman, preceded or succeeded by considerable velocity of pulse, which is seldom less than from 120 to 140 strokes in a minute; pain upon pressing the belly is soon complained of, with a degree of fulness and by and by tension. This pain will readily be distinguished from after-pains by its situation and permanency, and particularly by its being diffused over the whole external surface of the belly; so that after the disease is established, no one part of the abdominal surface can be touched ever so lightly without adding to the uneasiness; and, towards the close of the disease, even the pressure of the bed-clothes becomes troublesome. The appearance of the countenance is soon characteristic of unspeakable dejection and anxiety; and this symptom seems to me particularly to accompany this disease.

The proper treatment of so dangerous a disease as Puerperal Fever always proves itself, must be a point of first-rate importance to every gentleman interested in midwifery, since the very existence of his patient is wholly dependent upon his well timed exertions. The inherent powers of the human frame, so frequently successful in resisting the attack and progress of many diseases, are not to be relied upon in this; so that the conflict between those natural powers and the progressive ravages of Puerperal Fever must be decided by the judicious efforts of the medical art. What those efforts ought to be, and how and when, to be successful, they ought to be applied, have been so fully detailed by Dr. Gordon in his Treatise, that it might seem almost superfluous in me to say more than refer the reader to that pamphlet for ample information, had I not the judicious practice of a most eminent surgeon in corroboration of its propriety and utility. The grand points of the practice consist of free and copious bloodblood-letting, to twenty or twenty-four ounces, within a short period after the attack of the disease, and afterwards of most liberal purging. Mr. Hey (whose name stands so justly celebrated in the volumes of surgery) informs me, that the extraction of twenty or twenty-four ounces (the quantity thought sufficient by Dr. Gordon) is not always effectual to subdue the disorder. "I have lately," says he, "in two cases, and those not the most severe, bled three times in one day, and have taken in the whole thirty-seven or thirty-eight ounces of blood with complete effect; at the same time purging the patient freely." Mr. Hey further observes, "that the bleeding ought always to be performed on the first day of the attack." It must be understood that this practice is to be entered upon decidedly and resolutely within a few hours after the first symptoms of the disease, (the earlier after its attack the better) or it will most undoubtedly fail, and only hasten the destruction of the victim. If considerable symptoms of irritation and debility have shewn themselves, or if effusion into the abdominal cavity has taken place, such treatment must necessarily be unsuccessful and will get into disgrace. It is not, however, from bleeding a patient sparingly, or from a confined orifice, that any good is likely to result: blood must be taken away liberally and in a full stream, and I think a practitioner would be justified in opening a vein in both arms, that the full effect of bleeding might be accomplished, with the least general loss to the constitution. When the abstraction of blood is either not well-timed, or the operation itself is not properly managed, it would, perhaps, have been more to the credit of the art, if it had been entirely omitted; under such circumstances it cannot produce the expected relief. After liberal bleeding, the intestinal canal must be evacuated in the first instance by a full dose of calomel and jalap, and repeated dejections are afterwards to be encouraged by a solution of any of the neutral salts, with or without antimonials. The general principle will be assisted by the application of a number of leeches to the abdominal parieties, and the bleeding encouraged by warm stuphs. Repeated clysters injected warm into the rectum, with or without opiates, possess their advantages.

Let it not, however, be supposed, that the practice thus strenuously recommended will be successful in every instance. Some cases seem determinedly fatal from the first onset; in such, we can only regret the inefficacy of our best exertions, and, having performed our duty in properly using those means which reason and experience dictate, calmly submit to the eventful dispensations of Providence.

In

In the more protracted states of the disease, palliative means can only be resorted to according to the urgency of the symptoms, but they will seldom be productive of permanent advantage. After attention to the bowels, momentary relief may, perhaps, be obtained by the injection of a small quantity of warm fluid into the rectum occasionally with a proper quantity of opiate: the tension and pain of the belly may, possibly, be relieved by leeching, but as to the application of blisters, they have generally been thought to do more harm than good by the irritation they have produced. Bark and the stimulant plan have been universally found at Leeds prejudicial. Digitalis, given in such doses as to diminish the velocity of the pulse, seems to me to promise more than any other medicine; but I should not place much confidence in this valuable remedy, if bleeding freely had not been premised. As to the beneficial effects of camphor, opiates, antimonials, salines, &c. they appear by no means sufficiently proved as to demand any positive confidence in them; in short, if *nature* does not do a great deal for the patient, art can offer very little effectual service.

In any inflammatory disease, such as common enteritis, peritonitis, or peripneumonia, though the symptoms be ever so disguised by anomalous appearances, an able practitioner does not hesitate to bleed largely from a free orifice, and to repeat the bleeding according to the urgency of the symptoms; neither, if a puerperal woman was attacked with an inflammatory affection of the chest, would he think it right to neglect the same means of relief; ought, then, the puerperal state to preclude the use of that valuable remedy at the very onset of derangement, in that too frequently fatal disease, acute and rapid peritonæal inflammation; but, particularly, as it has been so repeatedly had recourse to with the most decided success? A celebrated surgeon observed to me, that in peritonitis we were frequently satisfied with merely obtaining a fallacious truce to the symptoms.

The attack of Puerperal Fever is, however, occasionally so very insidious, that it shall have made considerable progress before it attracts the attention of the patient, or is recognized by the medical attendant; but with common attention, it will seldom have made such advances, as to do away the necessity of that decisive treatment before mentioned: a double degree of vigilance will be required in every instance of *accouchement* when the disease is prevalent, not merely to recognize, but to detect the commencing symptoms, for reasons too obvious to mention.

There is another point regarding Puerperal Fever to which I wish to draw the attention of your readers; that is, respecting pecting the contagious nature of the disease. Dr. Gordon has positively, from his own observations, decided that question in the affirmative. Mr. Hey informs me, that he is not certain whether it is infectious or not; but that he thinks it right to act as if it were really so. This certainly is the conduct of a man of prudence: for my own part, besides the impression made on my mind by the relation of authors, as far as one solitary case can be supposed to be convincing, the fact I am going to relate speaks in the strongest terms: the nurse who attended upon the woman first taken ill, went directly from her bed-side to that of the second patient, who was her daughter, when she fell into labor; and observe, that almost as soon as that process is well finished, the second patient is attacked with the disease, and dies within twenty-seven hours from its commencement! I leave every one to make his own comment. If the disease be contagious, every means to prevent its communication from one to another should be adopted, as well as such as promise to destroy the infection itself; as fumigations, lime-washings, and proper care of the linen and bedding. Medical men and nurses will do well to keep in mind, that they may, possibly, convey the infection in their clothes, and pregnant women ought to be cautioned not to visit others under the disease. I am disposed to think from some late occurrences, that there is something extremely prejudicial to the health of women not puerperal, in the malignancy of child-bed fever; but my facts are yet so sparing, that I cannot speak positively upon the subject; I will merely observe, that one of the women in attendance upon the first patient, was within a very few days after her death seized with a most violent erysipelas of both legs, which proved obstinate in cure.

Since the preceding observations were made, I have seen a third case in the same village: this proved of a milder and less rapid kind than either of the former, and did not commence till fourteen days after delivery. Free bleeding and liberal purging were had recourse to before I saw the patient, that I had merely to sanction the proceedings: these means procured early relief, and the patient soon recovered.

I have also seen a case of inflammation of the uterus succeeding the use of instrumental means to hasten the delivery of a woman under Puerperal Convulsions, with an ill-formed pelvis. This woman had been bled liberally during the labor to relieve the convulsions: about twenty-four hours after delivery she was seized with rigor, quick pulse, and pain in the uterine tumor, followed by some tension of the belly. The next morning twenty ounces of blood were taken from a free orifice, and the bowels purged: considerable relief was experienced experienced by these means, but the night of the day following, there was a recurrence of the above symptoms, when the medical gentleman in attendance took away about fourteen ounces more, and kept up the intestinal evacuations: These means completely checked the disease, and the woman afterwards continued to do well. I have merely further to observe, that the appearance of the blood in the last bleeding was extremely sizy, and the crassamentum deeply cupped on its surface; whereas in the former one, though the blood was lost from a very free orifice, the crassamentum shewed but little of the sizy appearance. The same observation has been made with respect to the blood taken in Puerperal Fever; the blood drawn soon after the attack shall shew but little of the sizy appearance; whilst that taken some time after the disease has been established, shall shew these appearances, with the cup-like cavity on the surface of the crassamentum, in a high degree.

